# Vergleich von Beckenbodentrainingsmethoden zur Therapie weiblicher Harninkontinenz

**DOI:** 10.1007/s00120-025-02743-x

**Published:** 2025-12-08

**Authors:** R. Röthlisberger, Laila Schneidewind

**Affiliations:** 1 Inselspital Bern, Universitätsklinik für Urologie, Freiburgstrasse 37, 3010 Bern, Schweiz; 2 UroEvidence@Deutsche Gesellschaft für Urologie, Berlin, Deutschland

## Orginalpublikation:

Hay-Smith EJC, Starzec-Proserpio M, Moller B, Aldabe D, Cacciari L, Pitangui ACR, Vesentini G, Woodley SJ, Dumoulin C, Frawley HC, Jorge CH, Morin M, Wallace SA, Weatherall M (2024) Comparisons of approaches to pelvic floor muscle training for urinary incontinence in women. Cochrane Database Syst Rev. Dec 20;12(12):CD009508.

## Übersetzung

### Hintergrund.

Beckenbodentraining („pelvic floor muscle training“, PFMT) ist eine empfohlene Behandlung für weibliche Stress‑, Drang- und gemischte Harninkontinenz. Das Training variiert in der Art der Übung (Beckenbodenmuskeln kontrahieren mit und ohne andere Muskeln), der Dosis und der Durchführung (z. B. Umfang und Art der Betreuung).

### Ziele.

Bewertung der Wirkungen alternativer Ansätze (Übungsart, Dosis und Durchführung) des Beckenbodentrainings (PFMT) bei der Behandlung von Harninkontinenz (Stress‑, Drang- und gemischte Inkontinenz) bei Frauen.

### Suchmethodik.

Wir durchsuchten das Cochrane Incontinence Specialized Register (Stand: 27. September 2023; es enthält CENTRAL, MEDLINE, ClinicalTrials.gov und ICTRP der Weltgesundheitsorganisation), suchten per Hand in Zeitschriften und Konferenzberichten und überprüften die Referenzlisten der relevanten Artikel.

### Auswahlkriterien.

Randomisierte, quasirandomisierte oder cluster-randomisierte Studien zur weiblichen Stress‑, Drang- oder gemischten Harninkontinenz, bei denen ein Studienarm die PFMT einschloss und ein anderer einen alternativen Ansatz zur Art, Dosis oder Durchführung der PFMT verfolgte. Wir schlossen Studien mit Teilnehmenden mit neurologischen Erkrankungen oder mit schwangeren oder kürzlich entbundenen Frauen aus.

### Datensammlung und Datenanalyse.

Zwei Autoren bewerteten unabhängig voneinander die Eignung und die methodische Qualität der Studien mithilfe des Cochrane RoB1-Tools. Wir extrahierten und überprüften die Daten und klärten Unstimmigkeiten durch Diskussionen. Die Datenverarbeitung erfolgte wie im *Cochrane Handbook for Systematic Reviews of Interventions *(Version 6) beschrieben. Die Synthese wurde in den Interventionsuntergruppen abgeschlossen.

### Hauptergebnisse.

Dies ist eine Aktualisierung einer vorherigen Version. Die Analyse umfasste 63 Studien mit 4920 Frauen; die vorherige Version umfasste 21 Studien mit 1490 Frauen. Die Stichprobengröße reichte von 11 bis 362.

Bei den Teilnehmenden handelte es sich um Frauen im mittleren Alter (45 bis 65 Jahren), die an einer gemischten Stress- oder Belastungsharninkontinenz litten (46 Studien) und weder eine vorherige Inkontinenzbehandlung oder Beckenbodenoperation noch eine nennenswerte Beckenbodenfunktionsstörung hatten.

Die Studien wurden in Ländern auf der ganzen Welt, meist in Ländern mit mittlerem oder hohem Einkommen (53 Studien), durchgeführt.

Alle Studien hatten einen oder mehrere Arme, in denen „direkte“ PFMT eingesetzt wurde, definiert als wiederholte, isolierte, freiwillige Kontraktionen der Beckenbodenmuskulatur.

Die Studien wurden unterteilt in Vergleiche der Art des Trainings (27 Studien, 3 Untergruppen), der Dosis (11 Studien, 5 Untergruppen, 1 Studie ohne Daten) und der Durchführung (25 Studien, 5 Untergruppen).

Die Daten zur Lebensqualität bei Inkontinenz werden hier als primärer Endpunkt angegeben. Die Daten zu unerwünschten Ereignissen wurden narrativ zusammengefasst.

### *Vergleich 1: Art der Übung.*

Koordiniertes Training (Körperbewegungen mit gleichzeitiger Kontraktion der Beckenbodenmuskulatur) vs. direkte PFMT

Koordiniertes Training kann die Lebensqualität leicht verbessern (standardisierter mittlerer Unterschied (SMD) −0,22, 95 %-Konfidenzintervall (KI) −0,44 bis −0,01; I^2^ = 81 %; 8 Studien, 356 Frauen; Evidenz von niedriger Vertrauenswürdigkeit).

### Indirektes Training (Übungen, die keine Kontraktionen der Beckenbodenmuskulatur sind) vs. direkte PFMT.

Eine direkte PFMT kann die Lebensqualität mäßig verbessern (SMD 0,70, 95 %-KI 0,38 bis 1,02; I^2^ = 78 %; 4 Studien, 170 Frauen; Evidenz von niedriger Vertrauenswürdigkeit).

### Indirektes Training kombiniert mit direkter PFMT vs. direkte PFMT.

Die Kombination von indirektem Training mit direkter PFMT macht möglicherweise wenig bis keinen Unterschied in der Lebensqualität (SMD −0,08, 95 %-KI −0,26 bis 0,10; I^2^ = 33; 7 Studien, 482 Frauen; Evidenz von niedriger Vertrauenswürdigkeit).

### *Vergleich 2: Übungsdosis.*

PFMT mit Widerstandsgerät vs. PFMT ohne Widerstandsgerät

PFMT ohne Widerstandsgerät kann die Lebensqualität bei Inkontinenz leicht verbessern, aber die Evidenz ist sehr unsicher (SMD 0,22, 95 %-KI −0,04 bis 0,48; I^2^ = 32 %; 3 Studien, 227 Frauen; Evidenz von sehr niedriger Vertrauenswürdigkeit).

### Maximale Beckenbodenmuskelkontraktionen gegenüber submaximalen Beckenbodenmuskelkontraktionen.

Keine Daten gemeldet.

### PFMT an mehr Tagen pro Woche gegenüber PFMT an weniger Tagen pro Woche.

Eine PFMT an mehr Tagen pro Woche kann die Lebensqualität bei Inkontinenz deutlich verbessern (SMD −1,60, 95 %-KI −2,15 bis −1,05; 1 Studie, 68 Frauen; Evidenz von niedriger Vertrauenswürdigkeit).

### PFMT in aufrechter Körperhaltung gegenüber PFMT im Liegen.

Keine Daten gemeldet.

### *Vergleich 3: Durchführung einer Übungsintervention.*

PFMT unter Aufsicht in der Klinik vs. PFMT zuhause.

Die klinische Überwachung kann die Lebensqualität bei Inkontinenz leicht verbessern, aber die Evidenz ist sehr unsicher (SMD −0,30, 95 %-KI −0,65 bis 0,05; I^2^ = 89 %; 3 Studien, 137 Frauen; Evidenz von sehr niedriger Vertrauenswürdigkeit).

### Mehr Klinikerkontakt für PFMT-Supervision versus weniger Klinikerkontakt.

Keine verwertbaren Daten gemeldet.

### Einzelsupervision von PFMT vs. Gruppensupervision.

Individuell betreute PFMT führt wahrscheinlich zu einem geringen bis gar keinem Unterschied in der Lebensqualität (SMD −0,18, 95 %-KI −0,35 bis −0,01; I^2^ = 0 %; 5 Studien, 544 Frauen; Evidenz von moderater Vertrauenswürdigkeit).

### PFMT unter klinischer Aufsicht im Vergleich zur Aufsicht über E-Health (mobile App-Kommunikation mit Ärzten).

Die klinische Überwachung macht möglicherweise nur einen geringen bis keinen Unterschied in der Lebensqualität bei Inkontinenz, aber die Evidenz ist sehr unsicher (SMD −0,11, 95 %-KI −0,41 bis 0,19; 1 Studie, 173 Frauen; Evidenz von sehr niedriger Vertrauenswürdigkeit).

### PFMT-Anleitung über E-Health gegenüber schriftlicher Anleitung.

Die Bereitstellung von E‑Health kann die Lebensqualität leicht verbessern (SMD −0,21, 95 %-KI −0,43 bis 0,01; I^2^ = 25 %; 3 Studien, 318 Frauen; Evidenz von niedriger Vertrauenswürdigkeit).

### Unerwünschte Ereignisse.

In 9 Studien wurden Daten zu unerwünschten Ereignissen erhoben; bei 66 von 1083 (6 %) Frauen trat ein unerwünschtes Ereignis auf. Fast alle Ereignisse standen im Zusammenhang mit der Verwendung eines intravaginalen oder intrarektalen Trainingsgeräts. Die unerwünschten Ereignisse waren Scheidenausfluss, Schmierblutungen oder Unwohlsein.

### Beschränkungen der Evidenz.

Vier Hauptfaktoren haben unser Vertrauen in die Evidenz beeinflusst: 44 Studien wiesen ein unklares oder hohes Risiko für Selektionsverzerrungen auf; die Daten waren in einigen Untergruppen spärlich, da es nur wenige Studien gab: Studien, in denen die interessierenden Endpunkte nicht gemessen wurden oder Studien, die keine verwertbaren Daten lieferten; die Ergebnisse waren inkonsistent; und viele Studien waren klein (ungenau).

### Schlussfolgerung der Autoren.

Obwohl es Evidenz von niedriger bis moderater Vertrauenswürdigkeit gibt, dass einige PFMT-Ansätze besser sind als andere, gab es bei einigen nur geringe oder gar keine Unterschiede.

Die *7th International Consultation on Incontinenc *empfiehlt die PFMT als Erstlinientherapie für Frauen mit Harninkontinenz. Direkte PFMT (wiederholte, isolierte, freiwillige Kontraktionen der Beckenbodenmuskulatur) kann im Vergleich zu indirektem Training zu einer geringen Verbesserung der Lebensqualität bei Inkontinenz führen. Was die Verbesserung der Lebensqualität anbelangt, so kann die PFMT einzeln oder in einer Gruppe überwacht werden, da sie bei diesem Endpunkt wahrscheinlich wenig, bis keinen Unterschied macht. Viele Vergleiche wiesen eine Evidenz von niedriger oder sehr niedriger Vertrauenswürdigkeit auf, oft weil es nur eine Studie oder mehrere kleine Studien mit methodischen Einschränkungen gab.

Weitere, besser konzipierte und berichtete Studien, die PFMT-Ansätze direkt vergleichen, sind erforderlich, insbesondere Studien, die die Übungsdosis untersuchen.

## Kommentar

Das PFMT ist seit vielen Jahren Mittel der ersten Wahl der konservativen Therapie der weiblicher Harninkontinenz. Viele Leitlinien, einschließlich der 7th International Consultation on Incontinence (ICI; [1]) und Leitlinien der europäischen urologischen Fachgesellschaft (EAU; [2]), empfehlen PFMT als Erstlinientherapie bei Stress- und gemischter Harninkontinenz. Dennoch bleibt die Frage offen, welcher Trainingsansatz tatsächlich die besten Ergebnisse erzielt – und wie intensiv sowie strukturiert die Durchführung sein sollte.

Bei dem vorliegenden Cochrane Review handelt es sich um eine Arbeit aus dem Jahr 2024, welche die bereits vorliegenden Daten aktualisieren sollte. Dabei analysierten die Autoren 63 randomisierte Studien mit insgesamt 4920 Frauen. Im Vergleich zur vorherigen Version (21 Studien, 1490 Frauen) zeigt sich eine Erweiterung der Evidenzbasis. Die eingeschlossenen Studien decken verschiedenste PFMT-Ansätze ab: Unterschiede in der Art der Übungen, der Trainingsintensität (Dosis) und der Durchführung (individuell, Gruppe, E‑Health etc.). Der primäre Endpunkt war die Lebensqualität, gemessen über validierte Inkontinenzfragebögen.

Da es sich bei PFMT um ein Training handelt, ist der Erfolg maßgeblich von der korrekten Ausführung der Übungen sowie der regelmäßigen und konsequenten Durchführung abhängig. Durch die Technologisierung und Vernetzung kamen neue Möglichkeiten für die Anleitung und Kontrolle der physiotherapeutischen Übungen auf. In verschiedenen Studien konnte gezeigt werden, dass die Art und Weise (individuell, Gruppe, online etc.) keine signifikanten Unterschiede in der Effektivität des Trainings ergeben [3–5]. Dies spiegelt sich auch im vorliegenden Review wider. Die Art und Weise der Instruktion scheint keinen signifikanten Unterschied zu ergeben. Es ist jedoch zu erwähnen, dass Ansätze mit App/E-Health höchstwahrscheinlich die Adhärenz verbessern könnten. Außerdem stellen sie eine gute Alternative dar, zumindest unserer Einschätzung nach, um die begrenzten physiotherapeutischen Kapazitäten zu entlasten. Die Vielfalt der Ansätze – von Heimübungen über Physiotherapie bis zu App-basierten Programmen – spiegelt die klinische Realität wider, in der Motivation, Verständnis und Körperwahrnehmung der Patientinnen entscheidender sind als die Art des Trainings selbst.

Interessanterweise hat ein koordiniertes Training, das andere Muskelgruppen mit einbezieht (z. B. Rumpfstabilisation mit gleichzeitiger Beckenbodenkontraktion), im Vergleich zu reiner PFMT eine leichte Verbesserung der Lebensqualität gezeigt. Umgekehrt war eine reine PFMT dem „indirekten“ Training (z. B. allgemeine Fitnessübungen) überlegen. Zwischen direkter und kombinierter PFMT fanden sich keine signifikanten Unterschiede. In der Behandlung der Belastungsinkontinenz ist immer zu erwähnen, dass eine Reduktion von Übergewicht eine signifikante Verbesserung der Inkontinenz ergibt [6]. Ein Gewichtsverlust von nur 5–10 % kann bereits eine Reduktion der Inkontinenz um bis zu 50 % bewirken [7].

In Anbetracht dessen, wäre es erstrebenswert, sowohl die Stärkung der Beckenbodenmuskulatur anzustreben als auch generell eine Erhöhung der körperlichen Aktivität, was in einer Reduktion des Körpergewichts enden kann. Dies hätte einen zusätzlichen positiven Effekt auf die Inkontinenz. Leider wird der Aspekt der Gewichtsreduktion und dessen Einfluss, insbesondere auf die Belastungsinkontinenz, in der vorliegenden Cochrane-Arbeit nur unzureichend adressiert.

Die hier erhobenen Daten weisen eine hohe Heterogenität auf. Die Evidenzlage ist somit als begrenzt einzustufen. Entsprechend fordern die Autoren zu Recht besser konzipierte Studien mit standardisierter Definition der Trainingsintensität. Von Interesse ist in dieser Situation eine Dosis-Wirkungs-Beziehung, insbesondere die Frage nach der Häufigkeit und Intensität des Trainings. Würde man dies aus Studien bezüglich Krafttraining extrapolieren, könnte daraus eine Empfehlung mit einer initialen Phase von intensiverem Training zum Muskelaufbau gefolgt von einer Phase der Erhaltung resultieren [8]. Es ist jedoch unklar, ob diese Art des Muskeltrainings auf den Beckenboden applizierbar ist. Im klinischen Alltag ist auch ein möglichst langer Effekt, idealerweise lebenslänglich, gewünscht. In diesem Review war die Nachbeobachtungszeit in den meisten Fällen sehr kurz (< 3 Monate). Studien mit einer längeren Beobachtungsdauer und Dokumentation des Therapieerfolgs und Adhärenz der Patientinnen im weiteren Alltag wären erstrebenswert.

Was in der vorliegenden Übersichtsarbeit weiterhin nicht thematisiert wird, ist die zusätzliche Applikation von weiteren konservativen Therapiemöglichkeiten, wie z. B. eine Neuromodulation. In der aktuellen klinischen Praxis wird PFMT oft in Kombination mit einer Stimulation des N. pudendus und/oder des N. tibialis angewandt. Es gibt eine immer größer werdende Datenmenge, welche die kombinierte Therapie unterstützt bzw. favorisiert [9, 10]. Daher erscheint eine systematische Übersichtsarbeit inklusive Metaanalyse solcher Daten ebenfalls sinnvoll.

Kritisch an der vorliegenden Übersichtsarbeit ist anzumerken, dass bestimmte betroffene Patientengruppen ausgeschlossen worden sind. Zum einen Patientinnen mit neurologischer Grunderkrankung, also solche die, wie oben bereits diskutiert, von einer Kombination mit Neuromodulation besonders profitieren könnten. Aus diesem Grund wären hier weitere Studien bzw. Metaanalysen für die klinische Praxis von enormer Relevanz. Zum anderen wurden in diesem Cochrane Review lediglich Patientinnen bis zu einem Lebensalter bis 65 Jahren untersucht. Allerdings ist bekannt, dass gerade Ältere bzw. geriatrische Patientinnen häufig unter einer Inkontinenz leiden. Unserer Einschätzung nach bedarf es hier, v. a. durch den demographischen Wandel bedingt, ebenfalls an weiterer systematischer Aufarbeitung.

Methodisch handelt es sich bei dieser erläuterten Übersichtsarbeit um eine solide Arbeit ohne Restriktionen bzgl. der Sprache inkludierter Studien sowie des Suchdatums. Zusätzlich wurden detailliierte Subgruppenanalysen durchgeführt und die Arbeit ist weiterhin aktuell. Eine Ende Oktober 2025 durchgeführte Update-Suche nach den Kriterien dieses Cochrane Reviews konnte keinen Anhalt für Studien erbringen, die die Evidenzlage im Wesentlichen verändern. Kritisch anzumerken ist lediglich, dass zur Qualitätsbewertung der Studien das RoB1-Instrument verwendet wurde und nicht das aktuelle RoB2-Tool, das eine umfassende Aktualisierung des vorherigen Tools aus dem Jahr 2008 darstellt. Allerdings ist selbst eine dezidierte Metaanalyse auf die Qualität der inkludierten Studien angewiesen. Leider haben 44 Studien, die der vorliegenden Arbeit zugrunde liegen, ein unklares oder hohes Risiko für Selektionsverzerrungen. Deshalb sind folgende Implikationen der Autoren für die zukünftige Forschung besonders hervorzuheben: Es ist unerlässlich, dass die Forscher die aktuellen Standards für die Berichterstattung über Studienzusammenfassungen, Studien und Interventionsbeschreibungen vollständig erfüllen und dass die Herausgeber von Journalen konsequent verlangen, dass diese auch berichtet werden. Wenn z. B. die Intervention und der Vergleich nicht vollständig beschrieben werden, können Studien in systematischen Übersichtsarbeiten falsch klassifiziert oder ausgeschlossen werden. Die Verwendung von Protokollpapieren oder ergänzenden Online-Materialien kann Forschern dabei helfen, die erforderlichen Details anzugeben und damit langfristig eine bessere Evidenz für die klinische Versorgung unserer Patienten herzustellen.

Zusammenfassend ist Beckenbodentraining nach wie vor ein essenzieller Bestandteil in der konservativen Therapie bei Harninkontinenz. Die Methode der Instruktion scheint von untergeordneter Bedeutung zu sein. Eine zusätzliche Gewichtsreduktion wäre wünschenswert und weitere Studien mit Fokus auf Langzeitergebnisse und Kombinationstherapien sind notwendig.

## Fazit für die Praxis

- Trainieren erscheint wichtiger als die Trainingsart.
- Beckenbodentraining („pelvic floor muscle training“, PFMT) sollte weiterhin aktiv durch Urologen bei Harninkontinenz empfohlen und kontrolliert werden – idealerweise in Kooperation mit physiotherapeutischen Fachkräften.
- Die zusätzliche Empfehlung zur körperlichen Betätigung zur Gewichtsreduktion sollte unterstützend gegeben werden.
- Künftige Forschung sollte sich weniger auf den Vergleich minimal unterschiedlicher Protokolle konzentrieren, sondern eher auf die Umsetzung, Motivation und Langzeiteffekte im realen klinischen Setting.
- Zukünftige Forschung sollte sich ebenfalls auf spezielle Patientengruppen fokussieren, wie z. B. die geriatrische Population.
